# Synthesis and Hydrogen Storage Properties of Mg-Based Complex Hydrides with Multiple Transition Metal Elements

**DOI:** 10.1021/acsaem.4c02871

**Published:** 2025-04-17

**Authors:** Evans Pericoli, Alessia Barzotti, Raffaello Mazzaro, Romain Moury, Fermin Cuevas, Luca Pasquini

**Affiliations:** 1 Department of Physics and Astronomy “Augusto Righi”, University of Bologna, Bologna 40127, Italy; 2 Istituto per la Microelettronica e Microsistemi (IMM), Consiglio Nazionale delle Ricerche (CNR), Bologna 40129, Italy; 3 Centre National de la Recherche Scientifique (CNRS), Institut de Chimie et des Matériaux Paris-Est (ICMPE), University Paris-Est Creteil, Thiais 94320, France

**Keywords:** hydrogen storage, complex hydrides, reactive ball milling, temperature-programmed desorption, compositional tailoring, structural properties

## Abstract

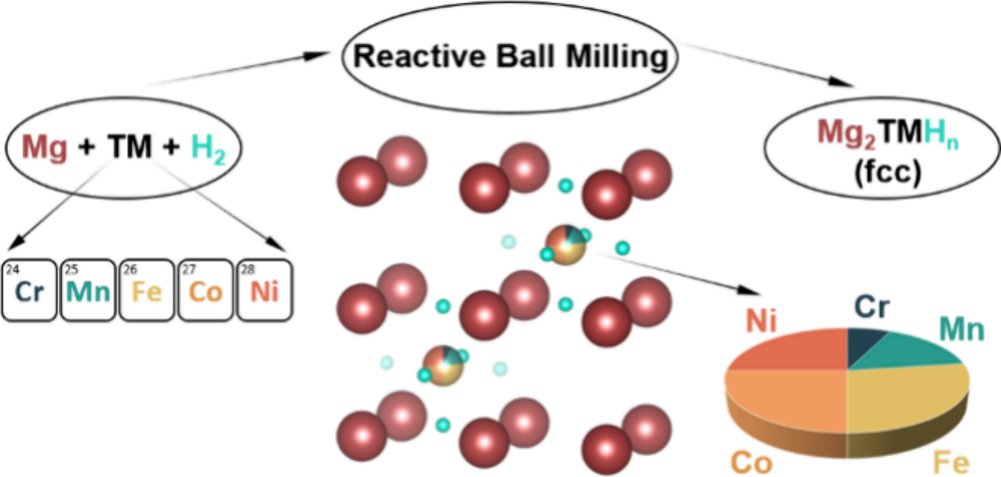

Mg_2_TMH_n_ complex hydrides, where TM represents various combinations of transition metals, were synthesized by reactive ball milling of Mg and TM powders under H_2_ pressure. TM was an equimolar mixture of three (Fe, Co, and Ni), four (Mn, Fe, Co, and Ni), or five (Cr, Mn, Fe, Co, and Ni) elements. The Mg/TM ratio was either 2:1 or 3:1. For 2:1 samples, a single fcc hydride phase Mg_2_TMH_n_ with a K_2_PtCl_6_-type structure was detected by X-ray diffraction along with a residual, unreacted metal phase. By contrast, in samples where the Mg/TM ratio was 3:1, the tetragonal MgH_2_ hydride was also observed. The formation of Mg_3_TMH_n_ complex hydrides, previously reported for TM = Cr and Mn under high-pressure conditions, was not detected. The maximum hydrogen content in the as-milled state was about 5 wt% for samples with a 3:1 Mg/TM ratio as determined by temperature-programmed desorption. The as-milled hydrides exhibited similar onset temperatures for desorption independently of the TM composition, suggesting no destabilization induced by elements like Mn and Cr that are known to form only unstable, high-pressure hydrides. The reversible hydrogen storage, investigated by pressure–composition isotherms in a Sieverts-type apparatus, arises from both the Mg-MgH_2_ and the Mg_2_TM-Mg_2_TMH_n_ transformations. Within the 0.1–20 bar and 285–320 °C window, the samples with a 3:1 Mg/TM ratio exhibit a reversible gravimetric capacity in the 3.7–4.2 wt% range depending on TM composition, while those with a 2:1 ratio are in the 3.0–3.2 wt% range. The decreased reversible capacity compared to the initial hydrogen content was associated with the phase segregation of the transition metals, particularly Cr and Mn, which was highlighted by X-ray diffraction and transmission electron microscopy with nanoscale microanalysis.

## 1 Introduction

In the ongoing search for novel, lightweight hydrogen (H) storage materials, solid-state Mg-based hydrides still offer promising solutions thanks to their low cost, high hydrogen storage capacity (7.6 wt%) and nontoxic nature, despite the high stability of MgH_2_ (which is conversely a favorable feature for heat storage in solar thermal power technologies^1^) and its relatively poor catalytic activity.^2^

Engineering the materials at the nanoscale can potentially address catalytic and stability limitations by refining microstructure, improving the physical coupling between complementary phases, and enhancing nanocatalytic effects for better hydrogen sorption properties.^3^ On the other hand, thermodynamic and kinetic properties can be tailored by introducing additives and substituents, which alter the microstructure and chemical environment, impacting the hydride stability and hydrogen uptake rate.^1,4^

Among these materials, Mg_y_TMH_n_ (where y = 2,3; TM = Cr, Mn, Fe, Co, Ni and n = 4 – 8) are of great interest due to their high gravimetric and volumetric densities and faster kinetics compared to pure MgH_2._^5,6^ The crystal structure of these ternary hydrides is based on the formation of complex anions that obey the 18-electron rule and show a strong covalent bond between H and TM^7^. In Mg_2_FeH_6_, the octahedral complex anion [FeH_6_]^4–^ is surrounded by eight Mg^2+^ cations in a cubic arrangement, and the crystal structure is a cubic K_2_PtCl_6_-type (space group *Fm*3̅*m*).^7−9^ In Mg_2_CoH_5_, the complex anion [CoH_5_]^4–^ has a square pyramidal structure and is surrounded by four Mg^2+^ cations. At room temperature, Mg_2_CoH_5_ has a tetragonally distorted CaF_2_-type structure (space group *P*4/*nmm*).^7,9−11^ In Mg_2_NiH_4_, the complex anion [NiH_4_]^4–^ has a tetragonal arrangement and the RT crystal structure is monoclinic (space group *C*2/*c*).^7,9,12^ Interestingly, both Mg_2_CoH_5_ and Mg_2_NiH_4_ undergo an allotropic transformation at high temperature (215 and 230 °C, respectively)^8,13^ yielding the same cubic structure as Mg_2_FeH_6._^14−16^

The hydrides with TM = Fe, Co, and Ni have a high enthalpy of decomposition (77.4, 86, and 64.5 kJ mol^–1^ H_2_ respectively),^17−19^ which results in *T* > 250 °C for H_2_ release at 1 bar pressure, similar to binary MgH_2_. The synthesis of Mg_2_FeH_6_ and Mg_2_CoH_5_ is challenging due to the absence of a solid solution between Mg and Fe or Co, significant differences in their vapor pressures, melting points, and the instability of the Mg_2_Fe and Mg_2_Co intermetallic compounds in their binary phase diagrams.^5^ Consequently, conventional metallurgical melting techniques are inefficient. First attempts to synthesize the Mg_2_FeH_6_ ternary hydride involved annealing Mg and Fe pellets under a hydrogen atmosphere (20–120 bar) at 450–520 °C, as reported by Didisheim et al.^20^

Today, reactive ball milling (RBM), which consists of performing mechanical milling under a high-pressure hydrogen atmosphere,^5^ is widely regarded as one of the most effective methods for synthesizing these complex hydrides. RBM also offers insights into the hydride formation process through in situ reaction monitoring, employing mechanical connections or telemetric sensors to transmit the pressure–temperature data.^21^ The first reports on the synthesis of Mg_2_CoH_5_^11^ and Mg_2_FeH_6_^22^ by RBM were published in the early 2000s. The synthesis of Mg_2_TMH_n_ (with TM = Fe, Co, or Ni) by RBM of elemental precursors was reexamined in 2011 by Zhang et al., who highlighted the existence of a single reaction path for all TMs,^21^ the first step of which consists in the formation of MgH_2_ catalyzed by the presence of TMs. The second step leads to the complex hydride and involves a further hydrogen uptake for TM = Co and Fe.

It must be noted that, at variance with Fe and Co, Ni forms the intermetallic compound Mg_2_Ni, allowing the synthesis of the Mg_2_NiH_4_ ternary hydride through RBM using either elemental powders or Mg_2_Ni as starting materials.^5,23^

The versatility of Mg_2_(Fe, Co, Ni) compounds is highlighted by the synthesis of the quaternary Mg_2_(FeH_6_)_0.5_(CoH_5_)_0.5_, where [FeH_6_]^4–^ and [CoH_5_]^4–^ complex anions coexist within the same compound. As reported by Deledda and Hauback,^10^ this enables the tailoring of hydrogen storage properties based on transition metal content. In recent studies, Polanski et al. investigated the effects of using steel scraps instead of pure iron in the hydride synthesis.^24,25^ They successfully synthesized Mg_2_(Cr,Fe,Ni)H_x_, demonstrating a hydrogen storage capacity at subambient temperatures as low as −50 °C. These findings^25^ emphasize the important role of alloying additives and synthesis techniques, suggesting that hydride formation can be effectively achieved using recycled materials.

In contrast to the TM = Fe, Co, Ni case, the hydrides with TM = Cr, Mn are very unstable: they form only at extremely high H_2_ pressure and are not suitable for near ambient storage applications.^26,27^ The hexagonal structure of Mg_3_MnH_7_ (space group *P*6_3_/*mmc*) consists of octahedral complexes, [Mn(I)H_6_]^5–^, surrounded by a distorted cubic environment of Mg^2+^ counterions,^26^ along with interstitial H^–^. In contrast,^27^ Mg_3_CrH_8_ has an orthorhombic structure (space group *Cmcm*)^27^ with [Cr(II)H_7_]^5–^ complexes in a pentagonal bipyramidal coordination environment, along with interstitial H^–^. The synthesis of Mg_3_MnH_7_ was initiated by pressing MgH_2_ and Mn pellets (2:1 molar ratio) in a multianvil assembly.^26^ The formation of hexagonal Mg_3_MnH_7_ was detected via in situ X-ray diffraction at 16 kbar and 520 °C, with a phase transition to orthorhombic Mg_3_MnH_7_ at 53.5 kbar and 550 °C. Similarly, the formation of orthorhombic Mg_3_CrH_8_ was observed at 50 kbar and 640 °C, with a high-temperature Mg_3_CrH_8_ phase forming at 750 °C. Despite these extreme conditions, significant quantities of unreacted CrH and MgH_2_ remained at the end of the process.^27^

As stated before, Mg_2_TMH_n_ complex hydrides with mixed transition metals have been studied with the aim to tune the hydrogen sorption properties and understand their link with structural parameters. This was typically done by combining two elements that form stable ternary hydrides, such as Fe with Co^10,28^ and Co with Ni^9^.

In this study, we undertake the synthesis of Mg-based complex hydrides by RBM of Mg with either three (Fe, Co, Ni), four (Mn, Fe, Co, Ni) or five (Cr, Mn, Fe, Co, Ni) different transition metal elements in equimolar amount, to explore if the modifications induced in the structure and composition impact the hydride thermodynamics and its gravimetric capacity. Furthermore, we examine the two different Mg:TM ratios of 2:1 and 3:1 that are typical of stable and high-pressure Mg-based complex hydrides, respectively. It will be shown that Mg_2_TMH_n_ forms in all cases, although the solubility of Cr and Mn in the TM site of the complex hydride is low, differently from Fe, Co, and Ni. Furthermore, the formation of Mg_3_TMH_n_ phases similar to high-pressure hydrides obtained with TM = Cr or Mn is not observed. To characterize the thermodynamic properties of the compounds, we integrate structural, microstructural, and compositional analyses with volumetric measurements using a Sieverts’ apparatus and temperature-programmed desorption (TPD) measurements.

## 2 Experimental Section

### 2.1 Hydride Synthesis

To investigate the effects of different transition metal mixtures and Mg:TM ratios on hydride properties, six compositions were synthesized, as reported in Table 1. The first sample, Mg_2_FeH_x_, was used as a reference for comparison with hydrides containing mixtures of Cr, Mn, Fe, Co, and Ni. While Fe and Ni are known to improve kinetic properties, Co typically brings Mg_2_FeH_6_ sorption processes to milder temperature conditions.^8,29^ The additions of Mn and Cr were intended to explore the possibility of synthesizing the unstable complex Mg_y_TMH_x_, which generally exhibits a higher gravimetric capacity than the above-mentioned compositions. Both Mg:TM ratios of 2:1 and 3:1 were investigated to explore the influence of varying Mg coordination with each TM.

**Table 1:** Total H_2_ Uptake Measured during Reactive Ball Milling and Total H_2_ Release Recorded at the End of Temperature Programmed Desorption Runs^a^

sample	H_2_ uptake in RBM (H/f.u.)	H_2_ uptake in RBM (wt%)	H_2_ release in TPD (H/f.u.)	H_2_ release in TPD (wt%)	onset T for H_2_ release in TPD (°C)
Mg_2_FeH_x_	6.2(9)	5.9(9)	5.4(2)	5.2(2)	267
Mg_2_(FeCoNi)_1/3_H_x_	4.0(6)	3.9(6)	4.3(1)	4.0(1)	275
Mg_2_(MnFeCoNi)_0.25_H_x_	4.2(6)	3.9(6)	4.9(2)	4.7(1)	274
Mg_3_(MnFeCoNi)_0.25_H_x_	6.6(9)	4.9(7)	6.4(2)	4.9(1)	257 (1st) 273 (2nd)
Mg_2_(CrMnFeCoNi)_0.2_H_x_	4.3(6)	3.8(6)	4.4(1)	4.2(1)	263
Mg_3_(CrMnFeCoNi)_0.2_H_x_	7.4(9)	5.5(8)	6.8(2)	5.3(2)	247 (1st) 277 (2nd)

a Notice that the H_2_ release in TPD expressed in H/f.u. represents the best estimation of the x value in pristine samples. The uncertainty of the onset temperature can be estimated as ± 3°C. The numbers in parentheses represent the standard deviation in units of the last significant digit.

The hydrides were prepared by Reactive Ball Milling (RBM) under 80 bar H_2_ pressure using a high-pressure milling vial by evico magnetics equipped with telemetric pressure–temperature sensors inserted in a Fritsch Pulverisette P6 planetary mill. The initial stoichiometries are reported in the first column of Table 1. For example, Mg_2_(FeCoNi)_1/3_ indicates a sample prepared with a proportion of 2 Mg + 1/3 Fe + 1/3 Co + 1/3 Ni. The synthesis started with fine powders of Mg (Alfa Aesar, 99.8%, 150 < Ps < 850 μm), Fe (Chempur, 99.9%, 74 < Ps < 149 μm), Co (Cerac, 99.8%, < 44 μm), Ni (Cerac, 99.8%, < 44 μm), and Mn (Cerac, 99.8%, < 44 μm). For samples Mg_2_(CrMnFeCoNi)_0.2_ and Mg_3_(CrMnFeCoNi)_0.2_, Mg powder was mixed directly with the presynthesized Cantor alloy (Cr, Mn, Fe, Co, Ni in equimolar fraction = 20%).

The vial, made of hardened steel, contained 41 stainless steel balls (12 mm diameter, 280 g in total) and 3 g of powder, resulting in a ball-to-powder ratio of 93:1. The syntheses were performed using 3 min milling cycles at 600 rpm followed by 9 min rest intervals, for a total of 100 cycles.

The radio-transmitted vial pressure and temperature were monitored throughout the process to calculate the total amount of hydrogen absorbed by the powder. During the initial cycles of each synthesis, the temperature increased due to heat generated by ball collisions and friction. After 1 h, it stabilized as a balance was achieved between heat generation and dissipation through the vial walls. Concurrently, the hydrogen pressure in the vial initially increased as the temperature rose. Then, it decreased due to the absorption processes (Mg/MgH_2_ and Mg_2_TM/Mg_2_TMH_n_) of the metallic powder. Due to temperature variations in the large volume of the vial (≈ 200 mL) and to the uncertainties in the pressure measurement, the relative uncertainties on the hydrogen uptake during RBM can be estimated as 15%. Typically, between 2 to 2.5 g of powders were obtained at the end of the milling cycles due to losses during the scraping of powders from the walls of the vial. The handling of the powders was conducted in an argon-filled purified glovebox.

### 2.2 Crystal Structure and Nanoscale Chemical Analysis

Powder X-ray diffraction (XRD) profiles were collected at ambient conditions using an Empyrean Panalytical Bragg–Brentano diffractometer equipped with the PIXcel^1D^ and PIXcel^3D^ multichannel solid-state detectors and a source of Cu K_α_ radiation. The diffraction patterns were analyzed via Rietveld refinement^30^ using the MAUD software.^31^ The instrumental function was determined from Rietveld refinement of a reference LaB_6_ powder. A few droplets of isopropanol were added to the samples inside the glovebox before exposing the hydrides to ambient air. This step prevented oxidation during transfer and measurements. Diffraction profiles were also collected after three PCI cycles to investigate the structural changes induced by hydrogen sorption cycles.

High resolution transmission electron microscopy (HR-TEM) and scanning transmission electron microscopy (STEM-HAADF) characterization was performed on a FEI Tecnai F20, operating at 200 kV and equipped with EDAX Phoenix spectrometer with ultrathin window detector for energy dispersive X-ray spectrometry (EDX). Isopropanol was deployed in this case, too, to handle the samples in ambient air and avoid oxidation during the transfer to the TEM chamber.

### 2.3 Hydrogen Sorption Properties

Temperature-Programmed Desorption (TPD) experiments were carried out in a custom setup equipped with calibrated volumes in the temperature range from 30 to 375 °C at a scan rate of 2 °C/min. The typical mass of the powder was 300 mg. The amount of hydrogen released from the sample was calculated from the pressure and temperature in the calibrated volumes using the van der Waals equation. The typical uncertainties are about 3%. The onset temperature for H_2_ release was determined by the tangent intersection method, fitting the linear H_2_ concentration drops obtained from the TPD profiles. Specifically, two linear fits were performed: the first to the start of the heating ramp, the second to the linear part of the desorption process at high temperature. Then, using the equations of the two best fits, the intersection point corresponding to the onset temperature was calculated. For the two samples with a Mg:TM ratio of 3:1, two consecutive desorption processes with different slopes were observed at high temperatures, resulting in two distinct onset temperatures. This procedure is illustrated for all samples in Figure S8.

Pressure–Composition-Isotherms (PCIs) were measured using a custom Sieverts-type apparatus equipped with a MKS Baratron 722A24MCD2FA pressure sensor, with a 0.5% accuracy on the readings and 20 bar range. The PCI curves were measured over the pressure range from 0.1 to 20 bar. Correspondingly, the temperature range was comprised between 285 and 320 °C. The typical mass of the sample was 500 mg, and the powder was introduced in the Sieverts reactor directly in the inert environment of the glovebox. The purity of H_2_ used throughout all measurements was 99.995%.

The number n that defines the stoichiometry of the complex hydrides Mg_2_TMH_n_ was determined from the total gravimetric hydrogen capacity measured by TPD or PCI using the equation:
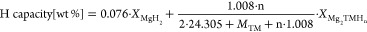
1Where *X*_MgH_2__ and *X*_Mg_2_TMH_n__are the phase abundances determined by Rietveld refinement expressed in wt%, 0.076 is the theoretical weight fraction of H in MgH_2_, 1.008 is the molar mass of H, 24.305 is the molar mass of Mg, and *M*_TM_ is the molar mass of the TM. The latter was determined using the molar mass of the elements and either the nominal starting composition or the local composition determined by STEM-EDX, when available (the difference between the two is not large because the TMs used are adjacent on the periodic table). The relative uncertainty on n is about 4–8% due to the propagation of uncertainties on phase abundancies and complex hydride composition.

On the other hand, the number x that defines the overall stoichiometry Mg_y_TMH_x_ of the pristine samples (neglecting impurities) is related to the capacity by
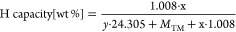
2Where y = 2,3 depending on the Mg:TM ratio in the starting powders. Therefore, the two numbers n and x used throughout this manuscript are clearly inter-related but have a different value and meaning.

## 3 Results and Discussion

### 3.1 Structure and Hydrogen Content of As-Milled Samples

**Figure 1:**
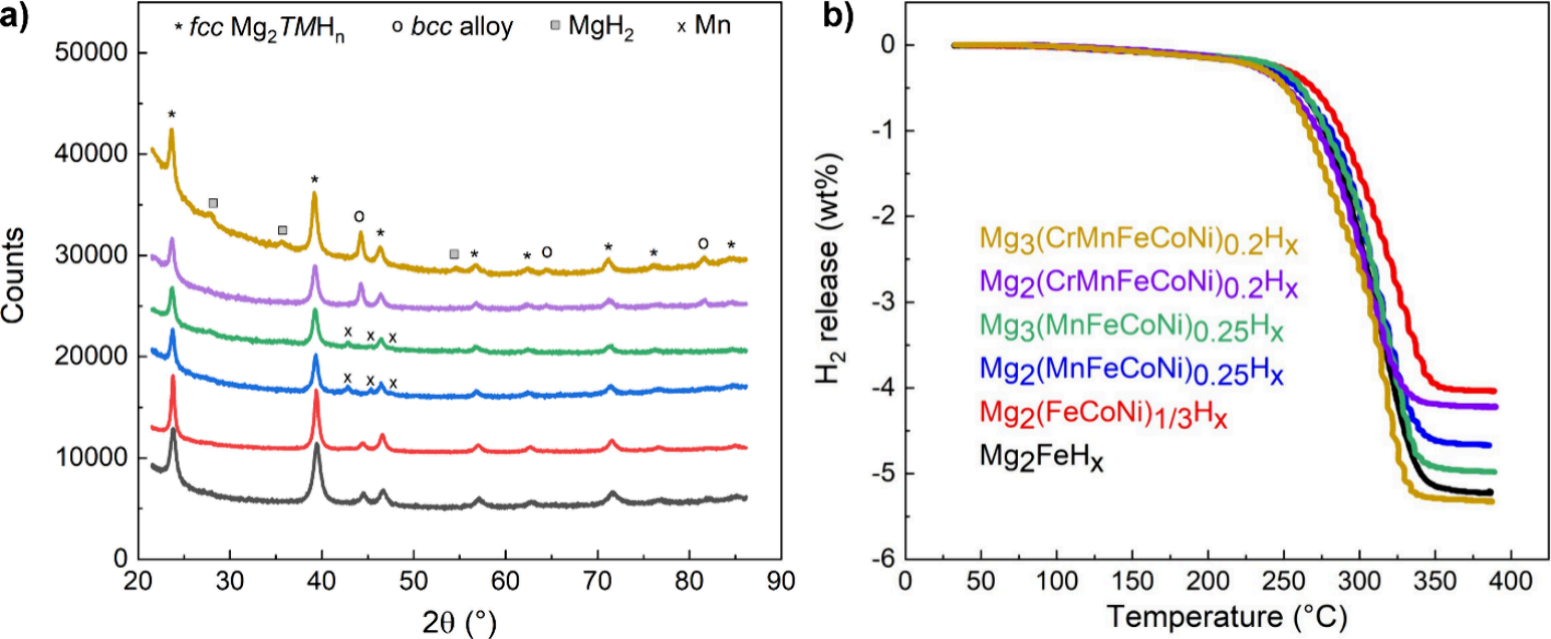
XRD profiles of as-milled powders (**a**); the profiles have been progressively shifted vertically for the sake of clarity. H_2_ release during TPD (**b**) reported in wt% (corresponding data in H atoms per formula unit in Table 1). The color code is the same in the two panels.

During reactive ball milling, substantial hydrogen absorption was recorded for all samples (Table 1 and Figures S9–S10). This absorption ranged from 3.8 to 3.9 wt% in samples where the Mg:TM ratio is 2:1 and TM is a combination of three or more elements, to 4.9–5.5 wt% for samples with a 3:1 Mg:TM ratio, and up to 5.9 wt% for the Mg_2_Fe starting mixture. These values correlate well with the observed formation of a high fraction (>90 wt%) of hydride phases, as demonstrated by the XRD profiles in Figure 1a and quantified by phase abundance analysis via Rietveld refinement (Table 2). All samples released H_2_ during TPD experiments, with desorption initiating above 250 °C and concluding by 350 °C (Figure 1b and Table 1). The amount of released H_2_ is compatible with the H_2_ uptake measured during the milling process within the experimental uncertainties, with TPD providing a more accurate determination. The total hydrogen content x in the pristine samples was calculated by eq 2 using the TPD-determined gravimetric capacity. The following paragraphs discuss the structural properties of as-milled samples in relation to their hydrogen content.

**Table 2:** Relative Phase Abundance (wt%), Lattice Parameter(s), and Stoichiometric Coefficient *n* of the fcc Mg_2_TMH_n_ Complex Hydride Phase Determined by Rietveld Refinement of XRD Profiles for As-Milled Samples^a^

sample	phases	wt%	a (Å)	c (Å)	n
Mg_2_FeH_x_	Mg_2_TMH_n_	95.5 (5)	6.442 (1)		5.7 (3)
	bcc alloy	4.5 (5)	2.872 (1)		
Mg_2_(FeCoNi)_1/3_H_x_	Mg_2_TMH_n_	86.8(4)	6.454 (1)		5.2 (2)
	bcc alloy	13.2(4)	2.880 (1)		
Mg_2_(MnFeCoNi)_0.25_H_x_	Mg_2_TMH_n_	90.2 (5)	6.468 (1)		5.5 (3)
	Mn (cubic *P*4_3_32)	9.8 (5)	6.320 (1)		
Mg_3_(MnFeCoNi)_0.25_H_x_	Mg_2_TMH_n_	88.6 (5)	6.471 (1)		5.0 (2)
	Mn (cubic *P*4_3_32)	3.1 (3)	6.321 (1)		
	MgH_2_	8.2 (4)	4.504 (1)	3.005 (1)	
Mg_2_(CrMnFeCoNi)_0.2_H_x_	Mg_2_TMH_n_	78.7 (5)	6.472 (1)		5.4 (2)
	bcc alloy	21.3 (5)	2.888 (1)		
Mg_3_(CrMnFeCoNi)_0.2_H_x_	Mg_2_TMH_n_	67 (1)	6.483 (1)		6.1 (3)
	bcc alloy	15.8 (6)	2.889 (1)		
	MgH_2_	17.2 (6)	4.516 (2)	3.019 (2)	

a The determination of *n* requires the use of TPD data (see eq 1 in section 2.3). The numbers in parentheses represent the standard deviation in units of the last significant digit.

**Figure 2:**
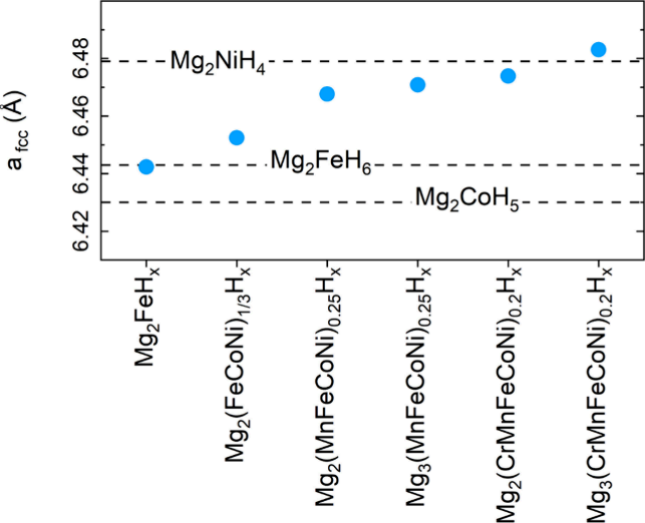
Lattice parameter of the fcc Mg_2_TMH_n_ phase determined by Rietveld analysis of XRD profiles. The dashed lines represent literature values for TM = Fe^20^, Co^19^, and Ni;^33^ the ones for TM = Co and Ni have been extrapolated to room temperature^10^ and corrected for the typical difference – usually around 0.01 *Å* – between hydrides and deuterides lattice parameters.

In each sample, the predominant phase exhibited the fcc crystal structure of K_2_PtCl_6_ (space group *Fm*3̅*m*); broad XRD peaks in Figure 1a indicate crystallite sizes of about 20 nm, characteristic of the RBM process. For clarity, this phase will be denoted as Mg_2_TMH_n_ in the following discussion of the TM composition and hydrogen stoichiometry n. We remind that n was calculated by eq 1 using the TPD-determined gravimetric capacity and differs from x in Mg_y_TMH_x_ because the samples are multiphase. The minimum lattice parameter *a* = 6.442(1) Å was observed for the Mg_2_FeH_6_ phase in the Mg_2_FeH_*x*_ sample (x = 5.4(2)) that contains also metallic Fe. This value matches well the values 6.443(1) Å^20^, 6.4403(5) Å^21^, and 6.4424(1) Å^32^ reported in XRD studies of Mg_2_FeH_6_ (see Figure 2). The corresponding value observed in deuterides is slightly smaller, 6.430 (1) Å.^8,20^Figure 2 also reports the reference values for cubic (space group *Fm*3̅*m*) Mg_2_CoH_5_ and Mg_2_NiH_4_: since these are high temperature phases, the literature values of 6.453 Å (Mg_2_CoD_5_ at 225 °C^19^) and 6.507(2) Å (Mg_2_NiD_4_ at 280 °C^33^) have been extrapolated to room temperature according to Deledda and Hauback^10^ and increased by 0.01 Å to account for the typical difference in lattice parameter observed between the hydride and deuteride phase. Looking at Figure 2, it is expected that the replacement of Fe with Co induces a slight contraction of the lattice parameter. This has indeed been reported by Barale et al.^8^ who carried out neutron powder diffraction on a series of Mg_2_Fe_*x*_Co_(1-x)_D_n_ complex hydrides (0 ≤ x ≤ 1 and n ≤ 5 ≤ 6). They showed that for Fe-rich compositions (x ≥ 0.5) only the cubic hydride phase is observed, while Co-rich compositions (x ≤ 0.1) crystallize in the tetragonal phase. The lattice parameter decreases by ≈0.01 *Å* (with a reported uncertainty of about 0.004 *Å*) with increasing Co content (from x = 1 to x = 0.5). On the other hand, Figure 2 shows that the replacement of Fe with Ni induces a larger expansion of the lattice parameter, which is about 0.036 *Å* larger in Mg_2_NiH_4_ compared to Mg_2_FeH_6_. Figure 2 also shows that the lattice parameter *a* increases as the number of elements in the starting mixture increases. By the light of the previous discussion, this result suggests a progressive change of the complex hydride composition, yielding a mixed character of the TM site.

**Figure 3:**
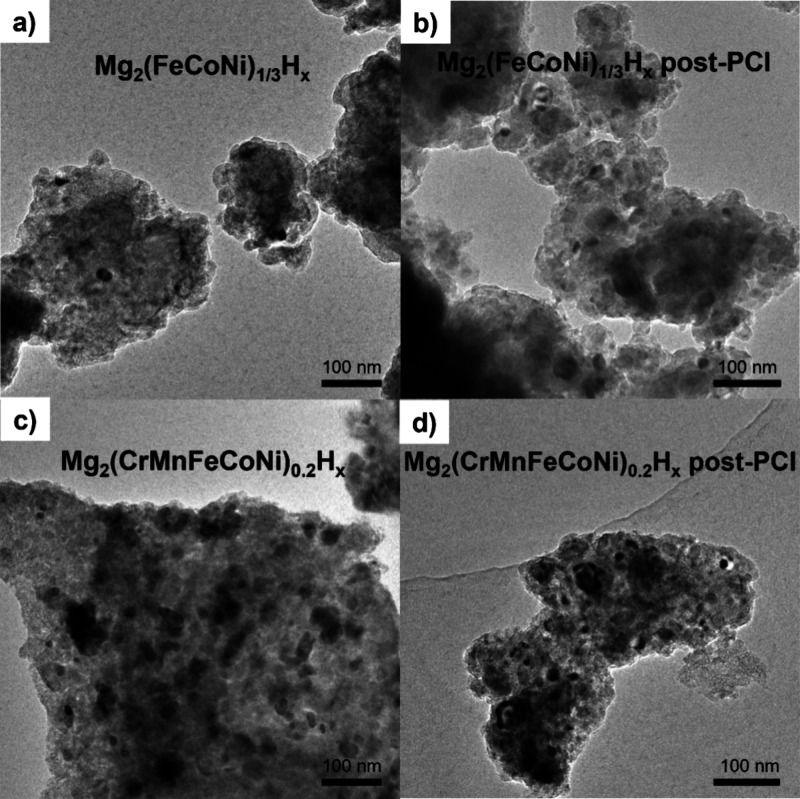
Bright field TEM images of Mg_2_(FeCoNi)_1/3_H_x_, Mg_2_(CrMnFeCoNi)_0.2_H_x_ (**a, c**), in the as-milled state and after the three absorption/desorption PCIs (**b, d**) shown in Figure 6.

**Figure 4:**
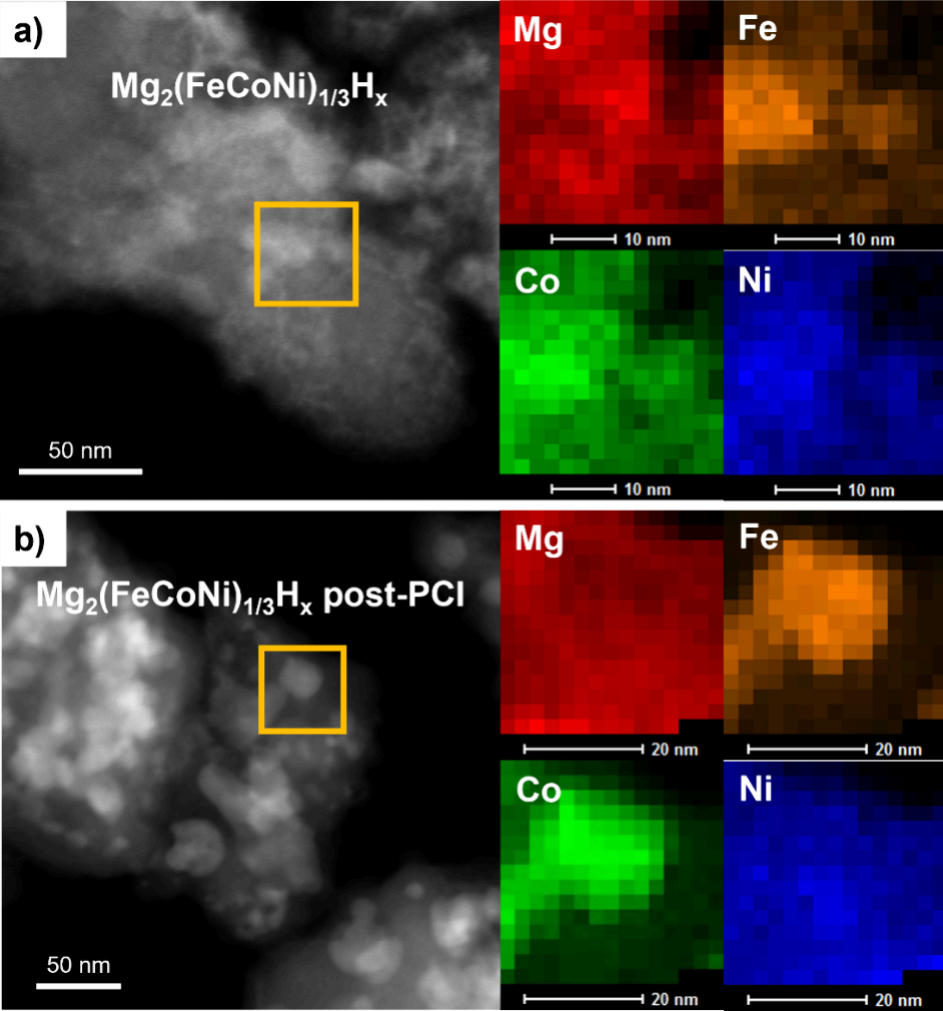
HAADF-STEM images and EDX maps of Mg_2_(FeCoNi)_1/3_H_x_ in the as-milled state (**a**) and after the three absorption/desorption PCIs (**b**) shown in Figure 6. The orange squares represent the area over which the mapping was collected.

**Figure 5:**
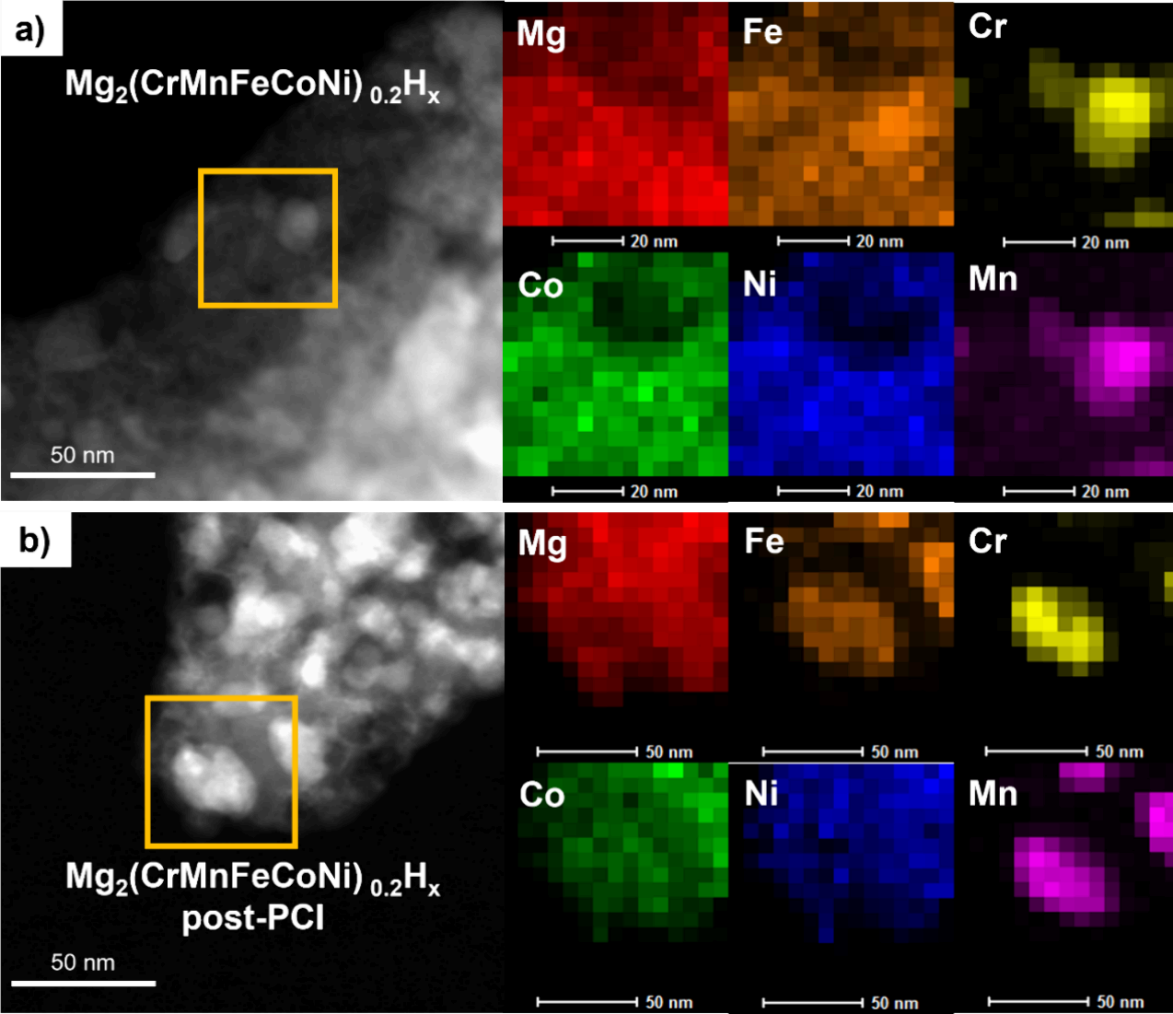
HAADF-STEM images and EDX maps of Mg_2_(CrMnFeCoNi)_0.2_H_x_ in the as-milled state (**a**) and after the three absorption/desorption PCIs (**b**) shown in Figure 6. The orange squares represent the area over which the mapping was collected.

Additional insights in this respect were obtained from TEM analysis (Figures 3–5). Bright-field TEM images (Figure 3a,c) show dark particles within a lighter matrix; these dark particles represent residual, unreacted metallic phases, while the light matrix is the Mg_2_TMH_n_ phase. The corresponding HAADF-STEM images (Figure S1a**,c**) and elemental mapping (Figures 4a and 5a) further characterize two selected samples, with quantitative EDX analyses of the matrix and metallic particles provided in Supplementary Figures S2a and S3a. In the Mg_2_(FeCoNi)_1/3_H_x_ sample (x = 4.3(1)), elemental mapping revealed that the Mg-rich matrix contains Fe, Co, and Ni in approximately equal amounts, suggesting the formation of an equimolar solid solution of Fe, Co, and Ni in the TM site of the Mg_2_TMH_n_ phase. The slightly larger lattice parameter in Mg_2_(FeCoNi)_1/3_H_x_ compared to Mg_2_FeH_x_ originates with the competing effects of Co and Ni^9^, as previously discussed. In fact, the expansion effect brought by Ni substitution is about three times larger than the contraction induced by Co (Figure 2), resulting in a net expansion when both Co and Ni replace Fe in the TM site in similar amount. In the Mg_2_(CrMnFeCoNi)_0.2_H_x_ sample (x = 4.4(2)), Cr and Mn were detected in small amounts within the Mg-rich matrix, while Fe, Co, and Ni were again present in similar molar ratios (Figure 5a and Figure S3a). This suggests limited Cr and Mn solubility in the TM site during milling, with most Cr and Mn atoms forming metallic particles embedded within the Mg_2_TMH_n_ phase, where TM sites are primarily occupied by Fe, Co, and Ni. This aligns with the absence of complex hydrides Mg_2_CrH_n_ and Mg_2_MnH_n_ reported in the literature. The presence of small amounts of Cr and Mn in the Mg_2_TMH_n_ matrix along with the increased lattice parameter compared to the case where TM = FeCoNi (Figure 2) suggests that Cr and Mn substitution induces a lattice expansion. A relevant comparison about the effect of Cr is the expanded lattice parameter *a* = 6.472 *Å* reported for Mg_2_(Fe,Cr,Ni)H_x_ prepared by ball milling of MgH_2_ and 316L austenitic stainless steel.^34^

Only samples with a 3:1 Mg:TM ratio exhibited broad reflections associated with tetragonal MgH_2_ (space group *P*4_2_/*mnm*)^4^. No evidence of a complex hydride with stoichiometry similar to Mg_3_TMH_n_, such as the high-pressure phases Mg_3_MnH_7_ or Mg_3_CrH_8_, was found. This indicates that the energy imparted during RBM is insufficient to promote the formation of high-pressure phases, as seen, for instance, in the RBM of MgH_2_, where high-pressure orthorhombic polymorphs can form alongside more stable tetragonal phases.^4,35^

A residual metallic phase was observed in all samples by XRD. This phase typically exhibited a bcc structure (space group Im3̅*m*) with a lattice parameter similar to Fe, except in samples where TM was an equimolar mixture of Mn, Fe, Co, and Ni, where it resembled the cubic *P*4_3_32 structure of Mn.^36^ This finding suggests that only part of the initial Mn enters the Mg_2_TMH_n_ phase as a solute in the TM sites. Specifically, the Mg_2_(MnFeCoNi)_0.25_H_x_ sample (x = 4.9(2)) showed a high weight fraction (9.8 wt%) of residual metallic Mn, indicating limited Mn solubility in the hydride. (S)TEM images (Figure 3a,c and Figure S1a,c) show metallic nanoparticles in the 10–50 nm range, consistent with crystallite sizes estimated from XRD broadening. In the Mg_2_FeH_*x*_ sample, the bcc lattice parameter *a* = 2.872 *Å* closely matches that of elemental bcc Fe (2.8665 Å),^37^ while larger lattice parameters were observed in mixed TM samples. For instance, in Mg_2_(FeCoNi)_1/3_H_x_ the metallic bcc phase had *a* = 2.880 *Å*, indicative of alloying effects from Co and Ni. This is corroborated by the STEM-EDX analysis (Figure 4a and Figure S2a), which identified the metallic particles as a Fe-rich FeCoNi alloy. Samples with an initial TM in the form of the Cantor equimolar alloy exhibited even larger lattice parameters (*a* = 2.888 *Å*), and STEM-EDX analysis (Figure 5a and Figure S3a) showed that these metallic alloys were rich in Cr and Mn, consistent with their low solubility in the Mg_2_TMH_n_ complex hydride.

The samples with a 3:1 Mg:TM ratio exhibited a two-step desorption process (Figure S8), attributed to sequential desorption from MgH_2_ and Mg_2_TMH_n_ phases. The onset temperatures of the second step (273–277 °C) match the onset range (263–275 °C) observed for single-phase desorption in samples with a 2:1 Mg:TM ratio, where only the Mg_2_TMH_n_ hydride is present. This suggests that H_2_ release above ∼ 265 °C originates from Mg_2_TMH_n_. The narrow onset range indicates similar thermal stability across different TM site compositions. Thus, forced Cr and Mn incorporation into the TM site during RBM does not significantly destabilize Mg_2_TMH_n_. The first desorption event in 3:1 Mg:TM samples, with an onset of 247–257 °C, is attributed to MgH_2_. Table 2 also summarizes the hydrogen stoichiometric coefficient n in as-milled Mg_2_TMH_n_. For Mg_2_FeH_*x*_, the value n = 5.7(3) aligns with the expected Mg_2_FeH_6_ stoichiometry. The reduction to n = 5.2(2) in sample Mg_2_(FeCoNi)_1/3_H_x_ is consistent with Co and Ni forming complex hydrides with lower hydrogen contents (5 and 4, respectively). The impact of Mn and Cr is less clear and no evident correlation with either the TM composition or lattice parameter can be identified within this limited set of data. Notably, determining n with high precision remains challenging due to cumulative uncertainties in H_2_ release and phase abundance measurements.

### 3.2 Reversible Hydrogen Capacity and Sorption Thermodynamics

**Figure 6:**
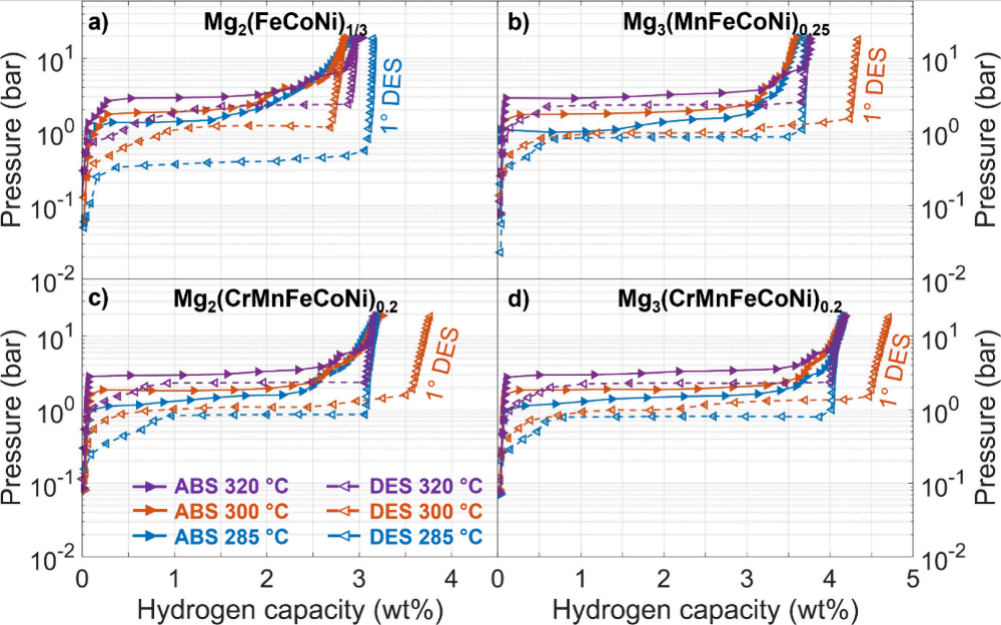
Absorption (ABS, right arrows, continuous lines) and desorption (DES, left arrows, broken lines) PCIs measured at 285, 300, and 320 °C for Mg_2_(FeCoNi)_1/3_ (**a**), Mg_3_(MnFeCoNi)_0.25_ (**b**)_,_ Mg_2_(CrMnFeCoNi)_0.2_ (**c**) and Mg_3_(CrMnFeCoNi)_0.2_ (**d**). Error bars within data points. The first desorption (indicated) started from as-milled powders; the last step was an absorption branch at 320 °C.

The as-milled powders were heated to either 285 or 300 °C under 20 bar H_2_, followed by a first desorption isotherm and an absorption isotherm at the same temperature. Additional desorption and absorption isotherms were performed in the 285–320 °C range, totaling three temperatures. This procedure was applied to four selected samples: two with a 2:1 Mg:TM ratio, and two with a 3:1 Mg:TM ratio. The pressure–composition isotherms (PCIs) are shown in Figure 6. Notably, the amount of H_2_ released in the first desorption isotherm from as-milled powders consistently exceeded the reversible H_2_ capacity observed in subsequent absorption–desorption cycles. This reduction in capacity is addressed in the next section, where the structural changes induced by cycling are examined. Compared to the TPD results, the H_2_ released during the first isotherm was slightly smaller. In three of the cases, the difference remained within approximately 0.5 wt%. However, for the sample Mg_2_(FeCoNi)_1/3_H_x_, this discrepancy was slightly larger. One possible explanation for this difference is a decrease in the stoichiometric coefficient n during the heating process to 300 °C under 20 bar, leading to unnoticed H_2_ release in the Sieverts equipment and thus to a lower H_2_ content. A reduction from the initial value n = 5.2 to n = 4.4 would be sufficient to explain the observed discrepancy in Mg_2_(FeCoNi)_1/3_H_x_. This observation suggests that n = 5.2 is a metastable stoichiometry obtained by RBM for TM = FeCoNi and that a partial H_2_ release occurs during heating, inducing a decrease of n toward the value typical for TM = Ni. Further experiments and verification would be required to confirm this point.

**Figure 7:**
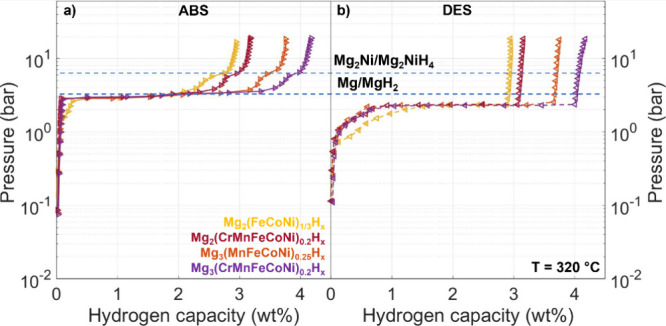
Comparison between absorption (**a**) and desorption (**b**) PCIs at 320 °C. The dashed lines denote the reference equilibrium pressure for the Mg_2_Ni/Mg_2_NiH_4_^18,21^ and Mg/MgH_2_^38,39^ transformation, as indicated. Error bars within data points.

The most notable difference between the samples is that those with a 3:1 Mg:TM ratio exhibit a higher reversible capacity (3.76–4.18 wt% at 20 bar, 320 °C) than those with a 2:1 Mg:TM ratio (2.95–3.19 wt% at 20 bar, 320 °C), see Table 3. The increased capacity is due to the presence of MgH_2_ following synthesis in the 3:1 samples, which is absent when the Mg:TM ratio is 2:1. MgH_2_ has a higher specific gravimetric capacity (up to 7.6 wt%)^4^ compared to any Mg_2_TMH_n_ phase.^21^ The equilibrium pressures at the three investigated temperatures remain constant across the compositions, as shown by the comparison of absorption and desorption isotherms at 320 °C in Figure 7. This observation suggests similar thermodynamics of H_2_ sorption across these samples, a finding also anticipated by the similar onset temperatures for H_2_ release observed in TPD analysis.

**Table 3:** Reversible Gravimetric Capacity (wt% H) Determined by Pressure–Composition Isotherms (0.1-20 bar Pressure Range, 320 °C), Enthalpy Δ*H* and Entropy Δ*S* of the Metal-Hydride Transformation (the Pressures Considered Being the Geometric Mean of the Absorption/Desorption 1st Plateau Pressures, See Eq 3)^a^

sample	reversible capacity (wt%)	Δ*H* (kJ mol^–1^ H_2_)	Δ*S* (J K^–1^ mol^–1^ H_2_)
Mg_2_(FeCoNi)_1/3_H_x_	2.95 (4)	74 (6)	132 (10)
Mg_3_(MnFeCoNi)_0.25_H_x_	3.76 (4)	75 (1)	135 (1)
Mg_2_(CrMnFeCoNi)_0.2_H_x_	3.19 (4)	72 (5)	129 (9)
Mg_3_(CrMnFeCoNi)_0.2_H_x_	4.18 (4)	74 (8)	133 (13)

a The numbers in parentheses represent the standard deviation in units of the last significant digit.

Figure 7 reveals a first absorption plateau at a pressure close to the reported value for the Mg/MgH_2_ transition,^38,39^ and a second plateau at a higher pressure characteristic of the Mg_2_Ni/Mg_2_NiH_4_ equilibrium.^18,21,40^ We thus attribute these two absorption plateaus to the formation of MgH_2_ and Mg_2_TMH_n_, respectively. Notably, the presence of the TM results in a more sloped second plateau. This effect may be ascribed to small fluctuations of Fe, Co, Ni, Cr and Mn content at the nanoscale, which correspond to a distribution of local compositions. Indeed, inhomogeneities in the chemical environment are typically responsible for the plateau sloping effect,^41,42^ as they introduce chemical potential gradients.

In the desorption isotherms, however, only a single plateau is observed - the slope seen at low H/M is a kinetic artifact due to the slow desorption kinetics near equilibrium, where the pressure was decreased before full equilibration. This suggests that both Mg_2_TMH_n_ and MgH_2_ release H_2_ at the same pressure. It has been proposed that the initial H_2_ desorption from one phase (here, Mg_2_TMH_n_, due to its lower stability) induces compressive strain in the other (MgH_2_), which raises the chemical potential of hydrogen and consequently its desorption pressure. This synergistic effect has been previously reported in Mg/Mg_2_Ni composite systems^43,44^ and may also apply to this nanoscale MgH_2_/Mg_2_TMH_n_ composite. In fact, as shown in the next section, MgH_2_ is present in all samples following the PCI cycles, even in those initially containing only Mg_2_TMH_n_.

From the geometric average of absorption (1st plateau) and desorption pressure, the equilibrium pressure at each temperature has been calculated according to
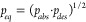
3

The use of a single equilibrium pressure instead of two separate values for absorption and desorption strongly reduces the errors due to pressure hysteresis. It is well-known that hysteresis causes an underestimation of the hydride formation enthalpy (in absolute value) and an overestimation of the hydride decomposition enthalpy obtained from Van ‘t Hoff analysis.^45−47^ In fact, the apparent discrepancy between these two values disappears when the enthalpy is determined by accurate calorimetric methods. Our procedure also reduces the experimental uncertainty because a double number of points is used for the construction of the Van ‘t Hoff plot.

**Figure 8:**
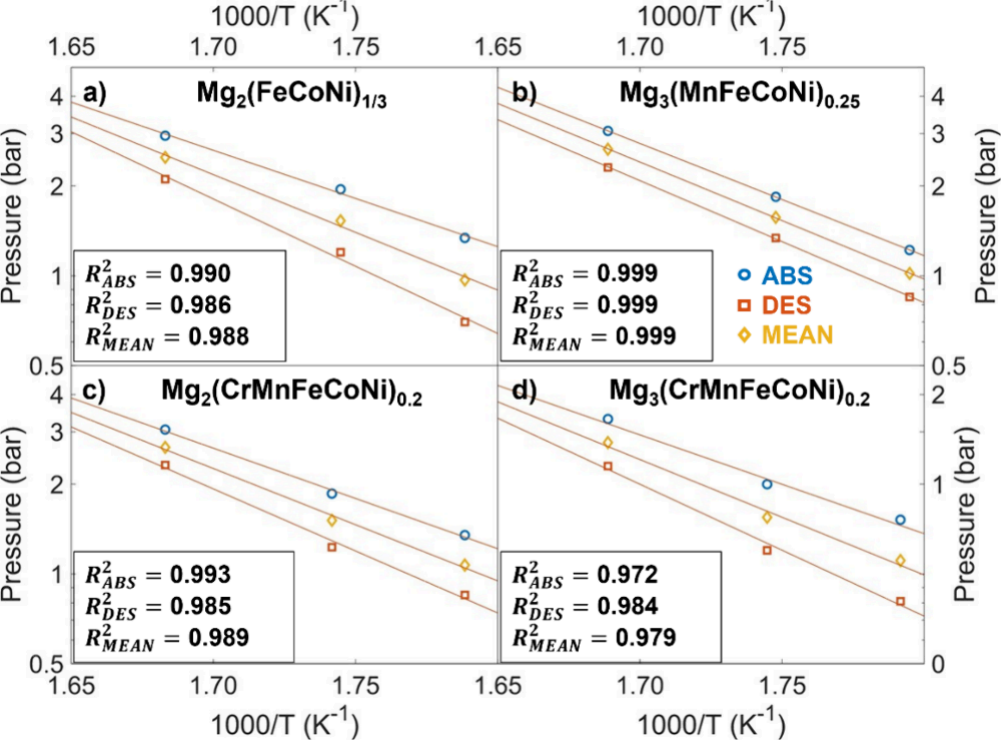
Van 't Hoff plots obtained from the PCIs of Figure 6 for Mg_2_(FeCoNi)_1/3_ (**a**), Mg_3_(MnFeCoNi)_0.25_ (**b**)_,_ Mg_2_(CrMnFeCoNi)_0.2_ (**c**) and Mg_3_(CrMnFeCoNi)_0.2_ (**d**). Yellow diamonds represent the geometric mean between absorption and desorption pressures.

The resulting Van ‘t Hoff plots are displayed in Figure 8, while the obtained enthalpy Δ*H* and entropy Δ*S* are listed in Table 3. These values, although affected by a large uncertainty due to the restricted temperature range, confirm the assignment of the first plateau to the Mg/MgH_2_ transformation, for which ΔH_dec_ = 74 kJ mol^–1^H_2_ and ΔS_dec_ = 133 J K^–1^ mol^–1^H_2_.^38,39^

### 3.3 Structural Changes Induced by Cycling

**Figure 9:**
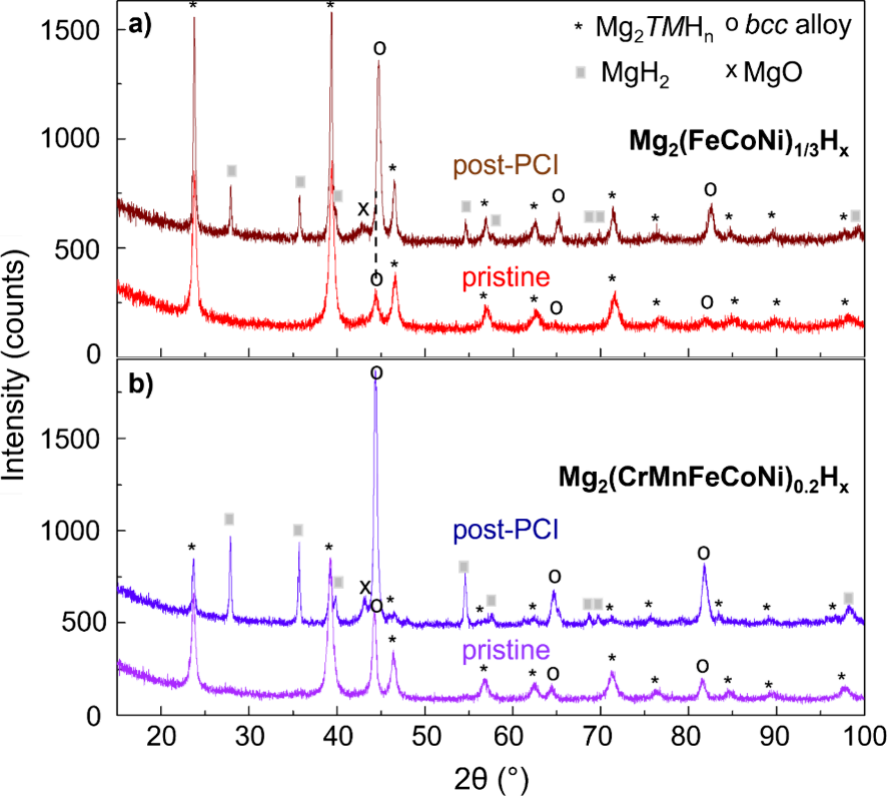
XRD analysis of the effect of hydrogen cycling on samples with 2:1 Mg:TM ratio and different TM compositions: FeCoNi (**a**) vs CrMnFeCoNi (**b**). The profiles labeled as “post-PCI” were collected after the absorption step at 320 °C of the third and last PCI.

The impact of hydrogen absorption/desorption cycles at elevated temperatures is highlighted by comparative XRD analysis (Figure 9) and (S)TEM-EDX examination (Figures 4b-5b). Quantitative phase abundances and lattice parameters are detailed in Table 4, with nanoscale EDX microanalysis provided in Figures S2 and S3. For the Mg_2_(FeCoNi)_1/3_H_x_ sample, an increase in the bcc alloy content is evident, rising from 13.2 wt% in as-milled powders to 29.5 wt% after three PCI cycles (Figure 9a, Table 4). An increase in the number of metallic particles is also clearly seen in the (S)TEM images (compare Figure 3b with a and Figure S1b with S1a). This result indicates that the hydride-formation process is not fully reversible, with a portion of the metal not reacting with Mg and H_2_ to reform the complex hydride Mg_2_TMH_n_. Consequently, excess Mg forms MgH_2_, as seen by the appearance of tetragonal MgH_2_ reflections in the XRD pattern. To identify which metals remain unreacted, EDX mapping (Figure 4b) and quantitative analysis (Figure S2b) were performed. Results show an increased atomic fraction of Co and Fe within the metallic particles and a corresponding decrease in the matrix, while Ni remains primarily in the Mg_2_TMH_n_ complex hydride (about 2/3 of the TM) and constitutes only 12% of the metallic particles. This finding is consistent with Ni ability to form compounds with Mg (such as Mg_2_Ni), which likely promotes its retention in the metallic state after desorption. Correspondingly, the lattice parameter of the fcc phase increases by 0.018 Å, trending toward the Mg_2_NiH_4_ value, while the bcc alloy lattice parameter decreases by 0.016 Å due to the reduced Ni content.

**Table 4:** Relative Phase Abundance (wt%), Lattice Parameter(s), Change of Lattice Parameter with Respect to the As-Milled Pristine Powders (Table 2 Data), and Stoichiometric Coefficient n of the fcc Mg_2_TMH_n_ Complex Hydride Phase Determined by Rietveld Refinement of XRD Profiles after Three PCIs on Selected Samples^a^

sample	phases	wt%	a (Å)	c (Å)	Δa (Å)	n
Mg_2_(FeCoNi)_1/3_H_x_	Mg_2_TMH_n_	45.4 (11)	6.472 (1)		+0.018	4.7(3)
	bcc alloy	29.5(7)	2.8640 (5)		–0.016	
	MgH_2_	11.5 (5)	4.516 (1)	3.021(1)		
	MgO	13.6 (8)	4.220 (2)			
Mg_3_(MnFeCoNi)_0.25_H_x_	Mg_2_TMH_n_	11.0 (9)	6.491 (1)		+0.021	5.0(3)
	bcc alloy	29.5 (5)	2.879 (1)			
	MgH_2_	42.3 (5)	4.520 (1)	3.023(1)		
	MgO	17.3 (10)	4.200 (1)			
Mg_2_(CrMnFeCoNi)_0.2_H_x_	Mg_2_TMH_n_	14.7 (3)	6.484 (1)		+0.012	5.3(4)
	bcc alloy	42.3 (5)	2.883 (1)		–0.005	
	MgH_2_	30.1 (5)	4.518 (1)	3.022(1)		
	MgO	12.9 (6)	4.200 (2)			
Mg_3_(CrMnFeCoNi)_0.2_H_x_	Mg_2_TMH_n_	12.6 (2)	6.483 (1)		0	5.5(4)
	bcc alloy	37.6 (4)	2.882 (1)		–0.007	
	MgH_2_	38.8 (4)	4.518 (1)	3.022(1)		
	MgO	13 (1)	4.212 (1)			

a The numbers in parentheses represent the standard deviation in units of the last significant digit.

In the sample with the initial TM composition as a Cantor alloy, even more pronounced changes are observed, with a significant increase in both the bcc alloy (from 21.3 to 42.3 wt%) and MgH_2_ content (Figure 9b, Table 4). This shift is due to a marked segregation of TMs into metallic particles, highlighted by (S)TEM images (compare Figure 3d-c and Figure S1d-c). STEM-EDX mapping (Figure 5b versus 5a) and quantitative microanalysis (Figure S3b) reveal that the complex hydride Mg_2_TMH_n_ is fully depleted of Mn and Cr, which instead dominate the metallic particles. Cr and Mn also appear to lower the Fe/Co ratio in the hydride, enriching the Fe content in the metallic particles (see maps in Figure 5b), likely due to differing alloying tendencies of Fe and Co with Mn and Cr. As observed with the previous sample, the lattice parameter of the Mg_2_TMH_n_ phase increases by 0.012 Å due to the higher Ni content in the TM sites, while the lattice parameter of the bcc alloy decreases slightly. Here too, Ni accounts for approximately 2/3 of the TM in the complex hydride.

For samples with a 3:1 Mg:TM ratio, phase analysis in Table 4 shows a similar trend, with increased metallic content and MgH_2_ abundance. In the sample where TM = MnFeCoNi, elemental Mn is absent after PCIs, indicating its alloying with Fe and Co in the metallic state following the initial desorption isotherm. Hydrogen sorption in these samples is largely dominated by the MgH_2_ ↔ Mg transformation, as MgH_2_ accounts for over 30 wt%, while Mg_2_TMH_n_ represents less than 15 wt%. The observed decrease in reversible gravimetric capacity from the initial desorption (Figure 6) is mainly attributed to the fact that the MgH_2_ + TM mixture has a lower capacity than Mg_2_TMH_n_ when n > 4. It is possible that residual metal reacts with Mg and H_2_ at pressures higher than those used in this study, potentially increasing the reversible capacity. However, this increase would likely be modest and might require additional high-pressure hydrogen handling beyond the levels typically achieved by modern electrolyzers.

We also notice that the XRD profiles of cycled samples reveal the formation of MgO with abundances that span the 13–17 wt% interval (Table 4), similar to previous reports on RBM Mg_2_TMH_n_ hydrides.^21^ These values are probably overestimated because of strong peak broadening, overlapping between MgO and other phases, and microabsorption of Cu K_α_ radiation by other phases that contain Fe. Nevertheless, the formation of MgO due to reaction of Mg with oxygen or moisture impurities adsorbed on the powders surface and/or present in the H_2_ gas causes a reduction of the hydrogen storage capacity. We notice that the crystallization of amorphous or ultrafine MgO, which may be present in the as-milled powders without being detected by XRD, would appear as an increase of the MgO content (which in fact is not) but does not cause a reduction of the capacity with cycling.

Across all PCIs in Figure 6, the reversible gravimetric capacity closely aligns with the H content estimated from the phase abundances in Table 4, assuming complete hydride-to-metal transformation for both MgH_2_ and Mg_2_TMH_n_. By inserting the PCI-determined gravimetric capacity and the XRD-determined phase abundances in eq 1, we estimate the stoichiometric coefficient n in the complex hydride, which is found to be approximately 5 for all samples (see Table 4). This estimation is qualitative, as uncertainties are significant due to the limited contribution of the Mg_2_TMH_n_ phase to total capacity, especially for the 3:1 Mg:TM samples.

## 4 Conclusions

Mg_2_TMH_n_ complex hydrides, where TM represents various combinations of transition metals, were successfully synthesized by reactive ball milling of Mg and TM powders under H_2_ pressure. The TM composition ranged from three elements (Fe, Co, Ni), to four elements (Mn, Fe, Co, Ni), and up to five elements using powders of Cantor alloy (an equimolar solid solution of Cr, Mn, Co, Fe, and Ni). Samples were synthesized with Mg:TM ratios of 2:1 and 3:1.

When the Mg:TM ratio was 2:1, only a single fcc hydride phase, Mg_2_TMH_n_, was obtained along with a residual, unreacted metal phase. By contrast, in samples where the Mg:TM ratio was 3:1, the tetragonal MgH_2_ hydride was also observed. The formation of Mg_3_TMH_n_ complex hydrides, previously reported for TM = Cr and Mn under high-pressure conditions, was not detected here. Cr and Mn proved challenging to incorporate into the Mg_2_TMH_n_ hydride structure; however, in as-milled samples using Cantor alloy, both Cr and Mn showed some solubility in the TM sites of the hydride, though this did not substantially destabilize the Mg_2_TMH_n_ phase even in the as-milled state. Indeed, H_2_ desorption from Mg_2_(MnFeCoNi)_0.25_H_x_ and Mg_2_ (CrMnFeCoNi)_0.2_H_x_ occurred at temperatures similar to Mg_2_FeH_6_ and Mg_2_(FeCoNi)_1/3_H_x_, which contain TM elements that readily form stable hydrides. Thus, the approach of destabilizing the hydride through Cr and Mn substitution in TM does not appear promising.

The residual metallic phase was found to be a bcc alloy in all cases, except for the as-milled samples with TM = MnFeCoNi, in which cubic Mn was identified. During hydrogen cycling, Cr and Mn – the elements that do not readily form stable Mg_2_TMH_n_ phases – were expelled from the Mg_2_TMH_n_ phase and segregated as metallic particles. Fe and Co exhibited similar behavior, albeit less prominently. As a consequence, the fraction of Ni in the TM site increased upon cycling, a feat that appears linked with its ability to form the stable intermetallic compound Mg_2_Ni. Following metal segregation out of the complex hydride upon cycling, MgH_2_ formation was observed even in samples with a 2:1 Mg:TM ratio. Samples with a 3:1 Mg:TM ratio displayed higher reversible gravimetric capacity (approximately 4 wt%) than the 2:1 samples, due to the greater presence of MgH_2_.

Thermodynamic analysis of hydrogen sorption, as determined by pressure–composition isotherms, showed two absorption plateaus corresponding to the MgH_2_ and Mg_2_TMH_n_ formation. The pressures for these plateaus aligned with literature values for these reactions. A single plateau was detected in the desorption isotherms, suggesting a synergistic effect where H_2_ release from one hydride phase facilitates release from the other through compressive stresses. The Van ’t Hoff analysis of the first plateau yielded enthalpy and entropy values consistent with those for the Mg-MgH_2_ system.

## Supporting Information Available

Dark field HAADF-STEM images; STEM-EDX images and microanalysis; XRD Rietveld refinement of selected hydrides; determination of the Onset temperature from TPD experiments; pressure and temperature evolution during RBM (PDF)
