# Supplementary material for: Synthesis and Hydrogen Storage Properties of Mg-Based Complex Hydrides with Multiple Transition Metal Elements
Source: ACS Appl Energy Mater. 2025 Apr 17;8(8):4993–5003. doi: 10.1021/acsaem.4c02871 (PMC12042823; doi:10.1021/acsaem.4c02871)
Supplement: Supplementary file 1 — ae4c02871_si_001.pdf [file ae4c02871_si_001.pdf]

Supporting Information

Synthesis and hydrogen storage properties of Mg-based complex hydrides with multiple transition metal elements

Evans Pericoli, [1] *Alessia Barzotti,* [1] *Raffaello Mazzaro*[1,2]*, Romain Moury,*[3] *Fermin Cuevas*[3] *and Luca Pasquini**[1,2]

[1] Department of Physics and Astronomy "Augusto Righi", University of Bologna, Bologna, 40127, Italy

[2] Istituto per la Microelettronica e Microsistemi (IMM), Consiglio Nazionale delle Ricerche (CNR), Bologna, 40129, Italy

[3] University Paris-Est Creteil, Centre National de la Recherche Scientifique (CNRS), Institut de Chimie et des Matériaux Paris-Est (ICMPE), Thiais, 94320, France

E-mail: luca.pasquini@unibo.it

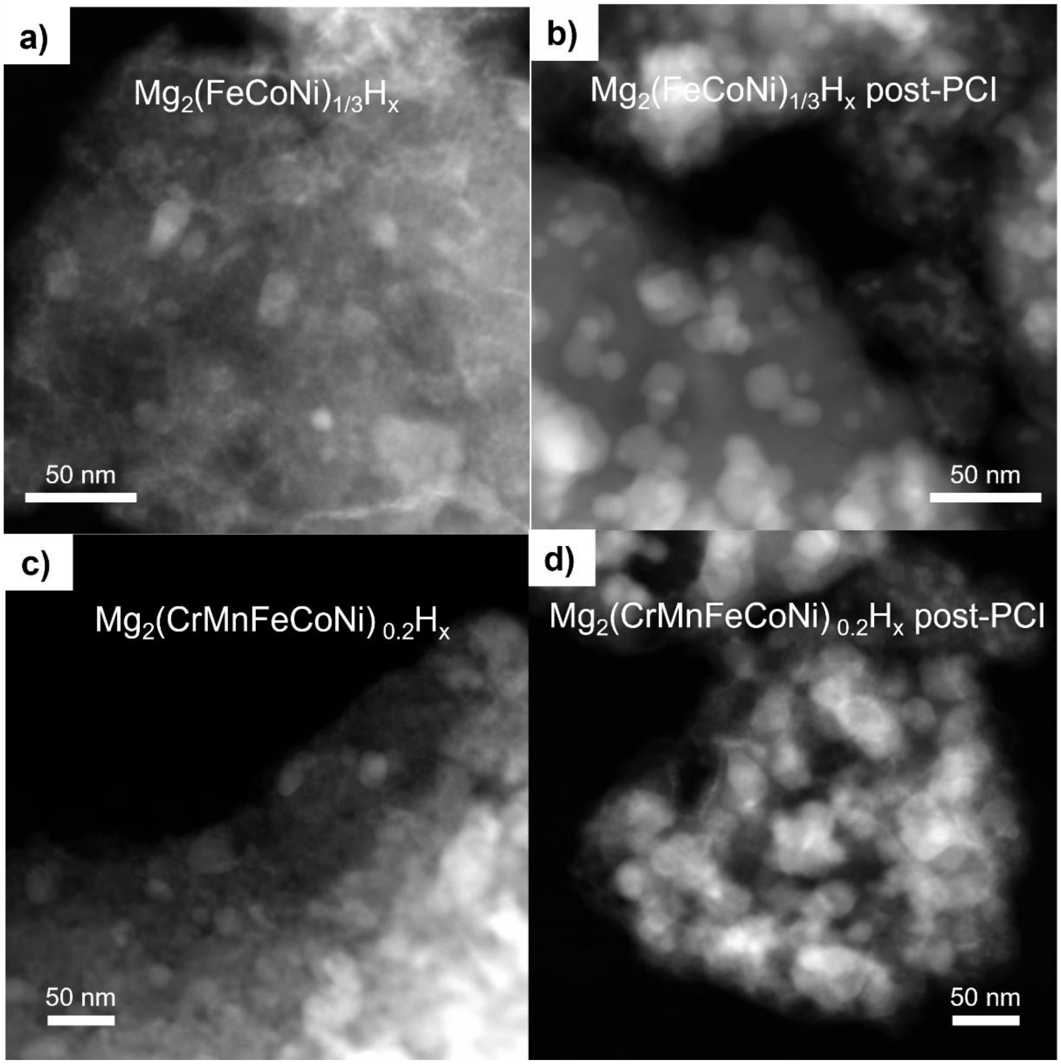

Figure S1: Dark field HAADF-STEM images of $Mg_2(FeCoNi)_{1/3}H_x$ and $Mg_2(CrMnFeCoNi)_{0.2}H_x$ in the as-milled state (**a,c**) and after the PCIs (**b,d**).

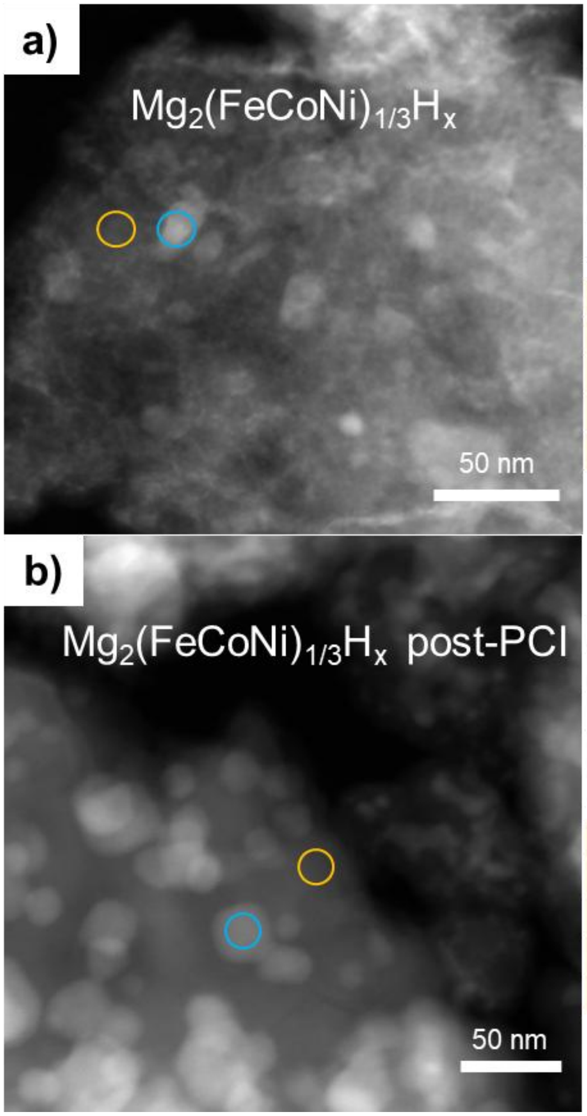

Element	Weight %	Atomic %
Mg(K)	12.5	25.12
Fe(K)	52.1	45.52
Co(K)	23.3	19.3
Ni(K)	12.1	10.05

Element	Weight %	Atomic %
Mg(K)	33.0	53.9
Fe(K)	22.6	16.1
Co(K)	24.5	16.5
Ni(K)	19.9	13.5

Element	Weight %	Atomic %
Mg(K)	11.7	24.0
Fe(K)	41.2	36.5
Co(K)	36.0	30.2
Ni(K)	11.0	9.3

Element	Weight %	Atomic %
Mg(K)	47.7	68.6
Fe(K)	7.4	4.6
Co(K)	9.4	5.6
Ni(K)	35.4	21.1

Figure S2: STEM-EDX quantification of $Mg_2(FeCoNi)_{1/3}H_x$ in the as-milled state (**a**) and after the PCIs (**b**).

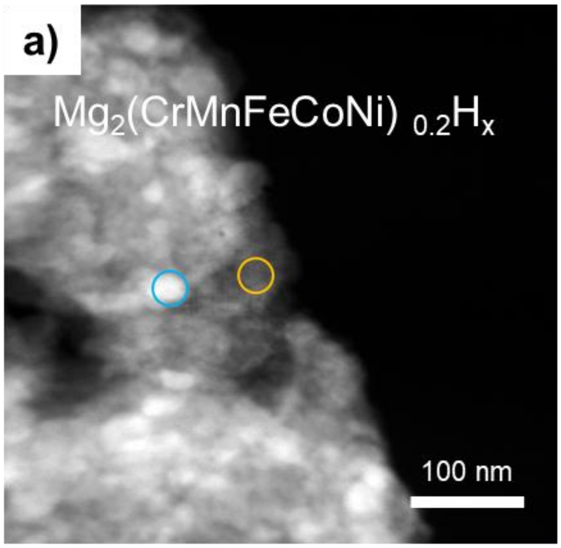

Element	Weight %	Atomic %
Mg(K)	14.73	27.89
Cr(K)	35.7	31.6
Mn(K)	22.03	18.46
Fe(K)	12	9.88
Co(K)	8.62	6.73
Ni(K)	6.89	5.41

Element	Weight %	Atomic %
Mg(K)	35.32	56.14
Cr(K)	3.99	2.97
Mn(K)	9.7	6.82
Fe(K)	17.02	11.77
Co(K)	17.3	11.34
Ni(K)	16.63	10.94

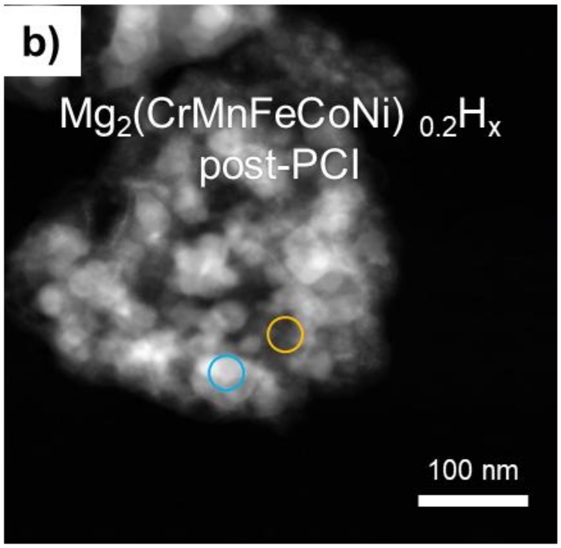

Element	Weight %	Atomic %
Mg(K)	29.18	48.14
Cr(K)	26.74	20.62
Mn(K)	16.97	12.39
Fe(K)	10.18	7.31
Co(K)	6.73	4.58
Ni(K)	10.16	6.94

Element	Weight %	Atomic %
Mg(K)	53.94	73.72
Cr(K)	0	0
Mn(K)	1.26	0.76
Fe(K)	6.35	3.78
Co(K)	10.19	5.74
Ni(K)	28.24	15.98

Figure S3: STEM-EDX quantification of $Mg_2(CrMnFeCoNi)_{0.2}H_x$ in the as-milled state (**a**) and after the PCIs (**b**).

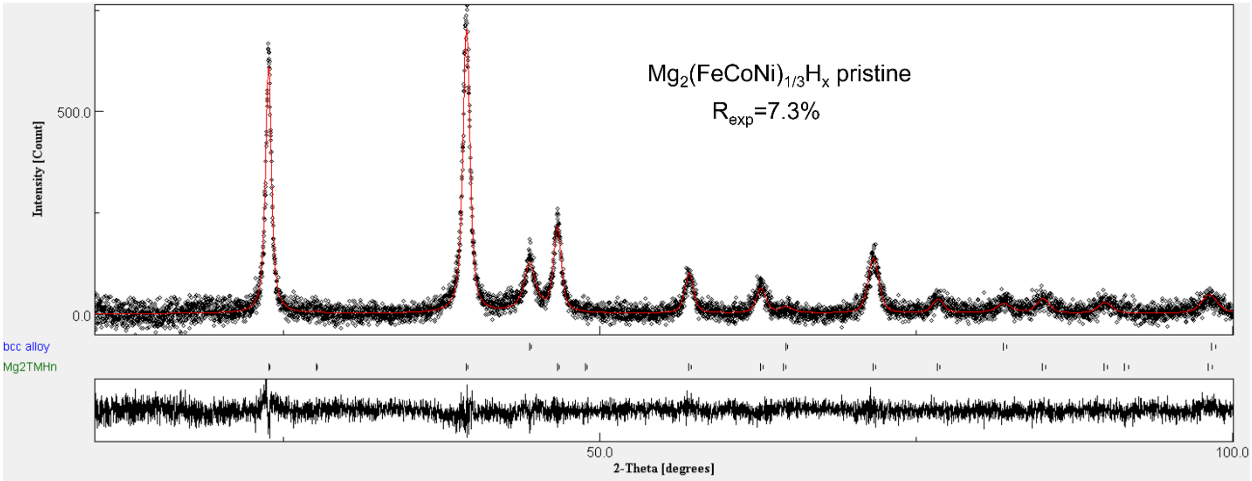

Figure S4: XRD profile and Rietveld refinement of the as-milled $Mg_2(FeCoNi)_{1/3}H_x$.

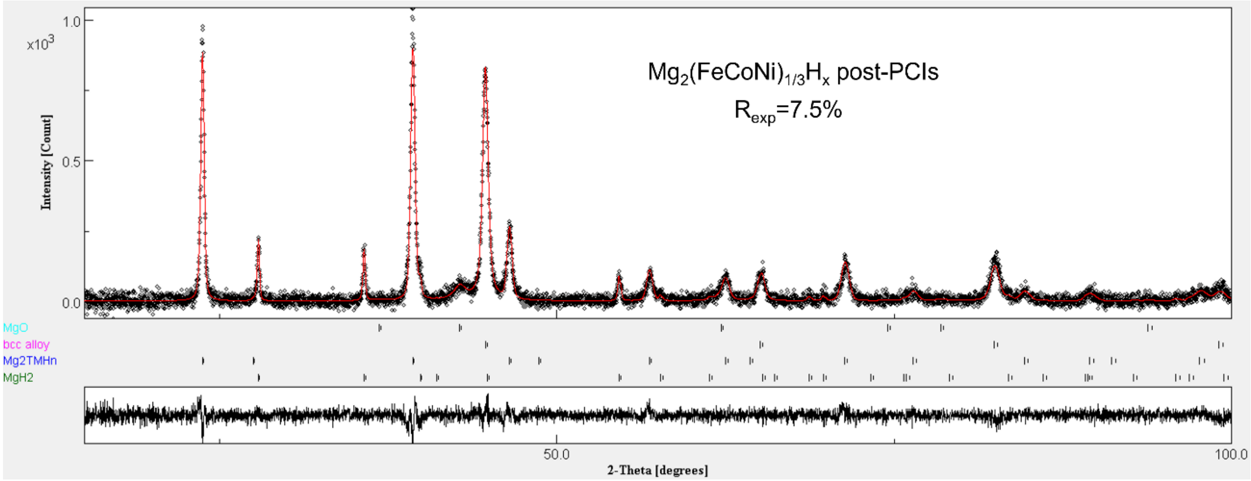

Figure S5: XRD profile and Rietveld refinement of $Mg_2(FeCoNi)_{1/3}H_x$ after PCIs.

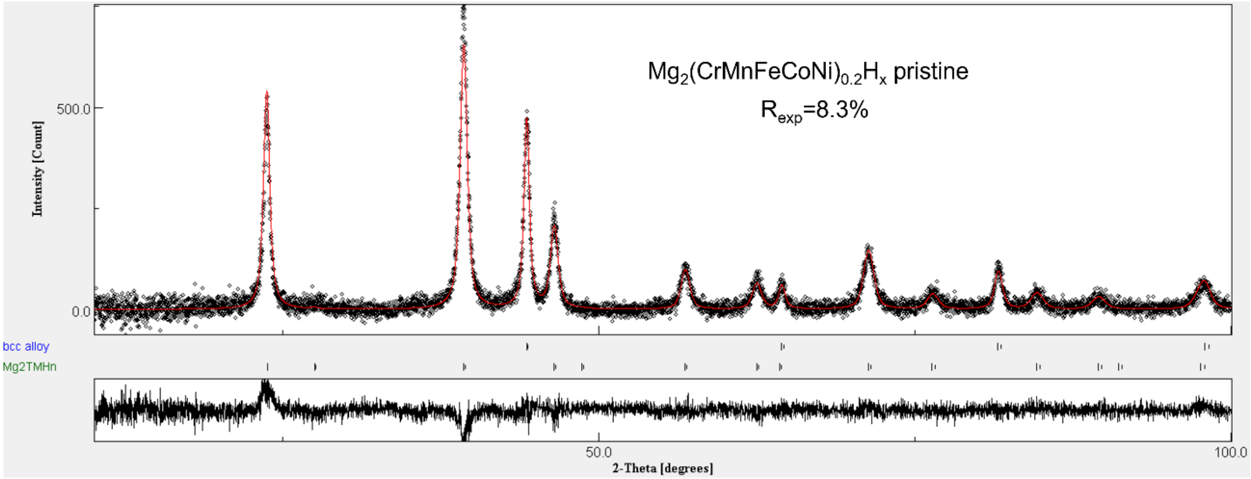

Figure S6: XRD profile and Rietveld refinement of the as-milled $Mg_2(CrMnFeCoNi)_{0.2}H_x$.

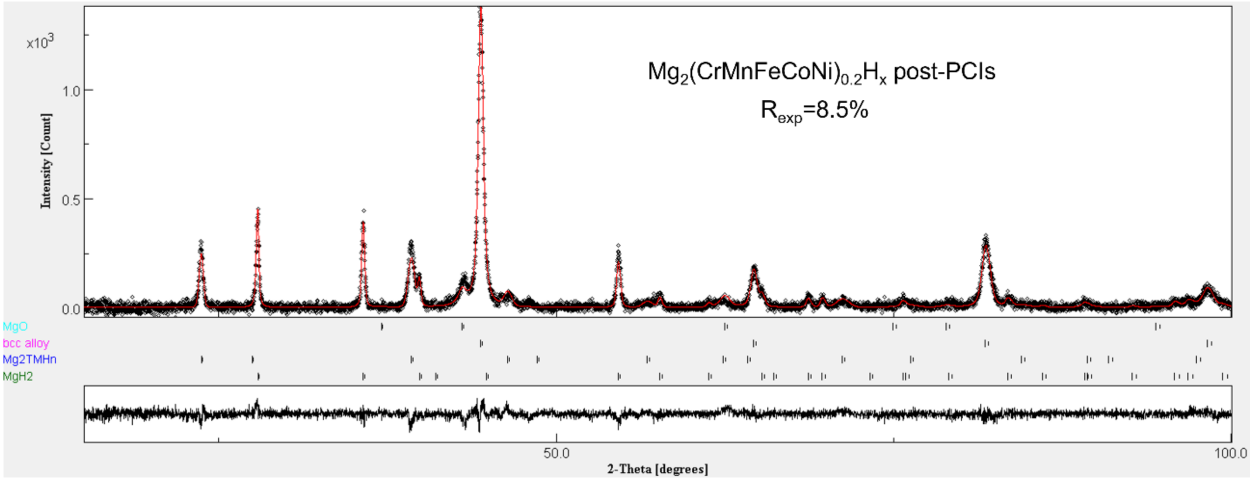

Figure S7: XRD profile and Rietveld refinement of $Mg_2(CrMnFeCoNi)_{0.2}H_x$ after PCIs.

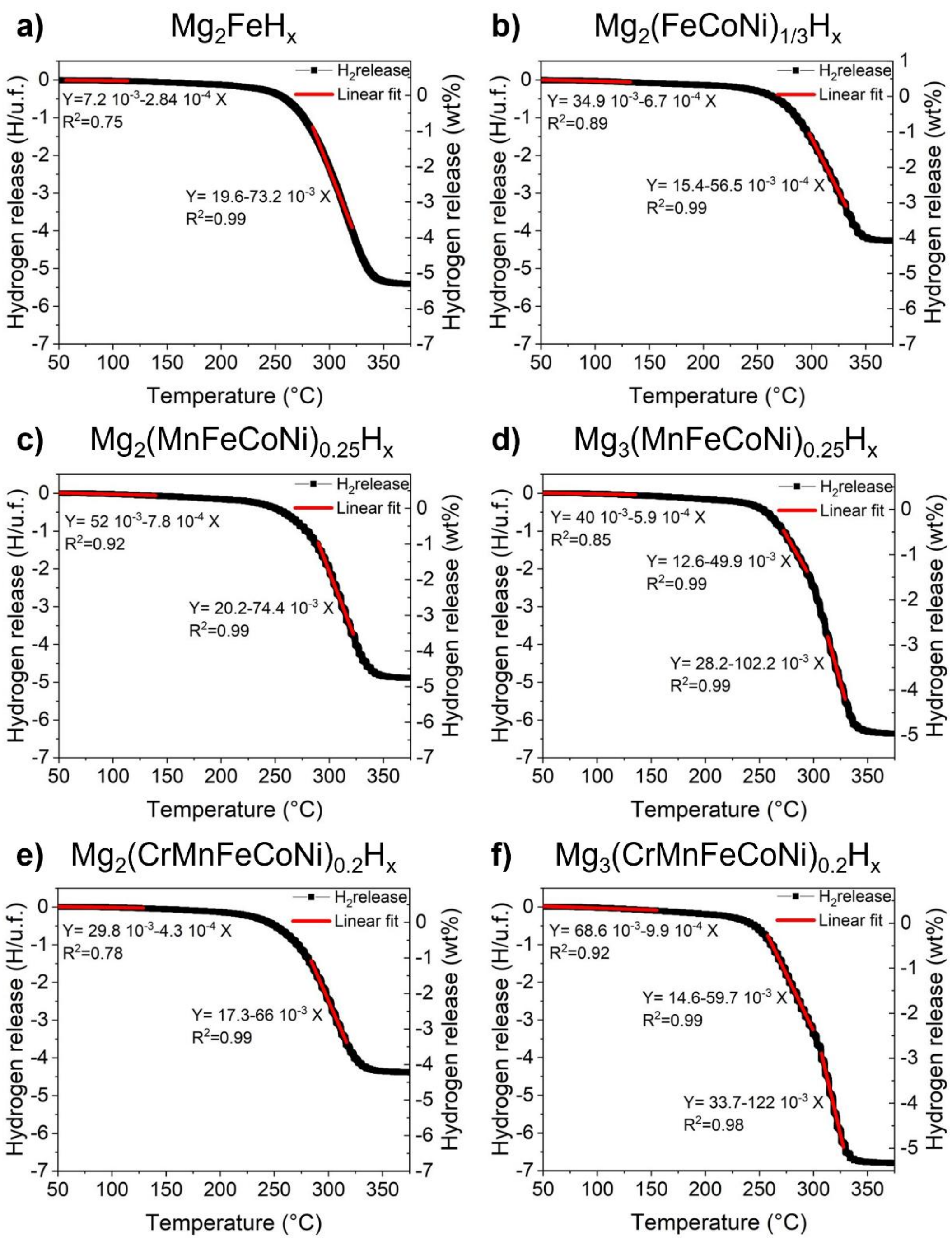

Figure S8: Determination of the onset temperature in TPD experiments by the intersection of linear fits to the ramp start and to the linear decreasing trends that represent H_2 release for Mg_2FeH_x (**a**), $Mg_2(FeCoNi)_{1/3}H_x$ (**b**), $Mg_2(MnFeCoNi)_{0.25}H_x$ (**c**), $Mg_3(MnFeCoNi)_{0.25}H_x$ (**d**), $Mg_2(CrMnFeCoNi)_{0.2}H_x$ (**e**), $Mg_3(CrMnFeCoNi)_{0.2}H_x$ (**f**).

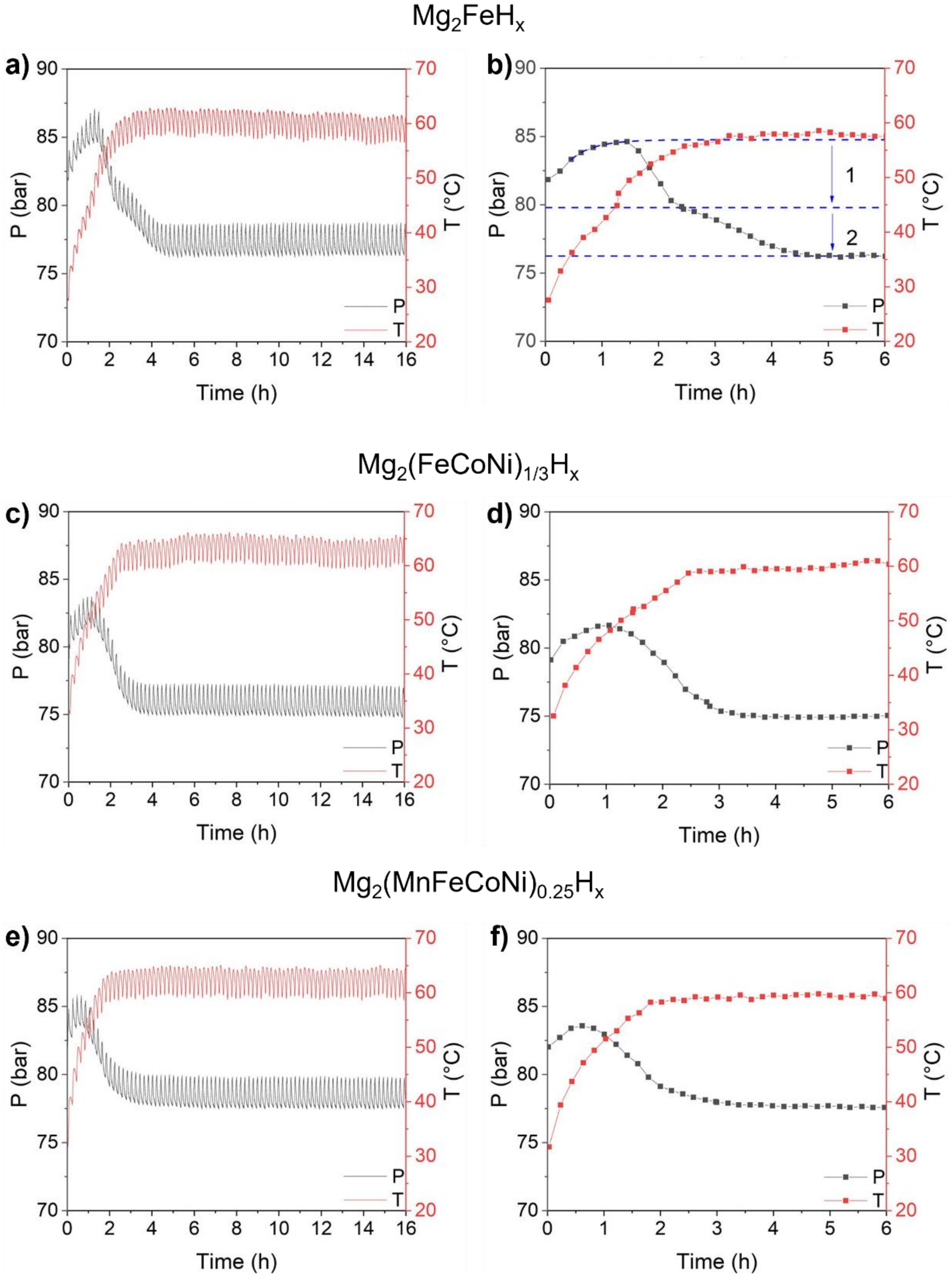

Figure S9: Pressure and temperature time-profile of the RBM synthesis process. Raw data of the full process (left) and p-T points collected at the end of each three-minutes cycle for Mg_2FeH_x (**a,b**), $Mg_2(FeCoNi)_{1/3}H_x$ (**c,d**), $Mg_2(MnFeCoNi)_{0.25}H_x$ (**e,f**). The blue arrows and dotted lines highlight the two-step process (Mg/MgH_2 and MgH_2/Mg_2TMH_2) the metallic mixture undergoes during the synthesis.

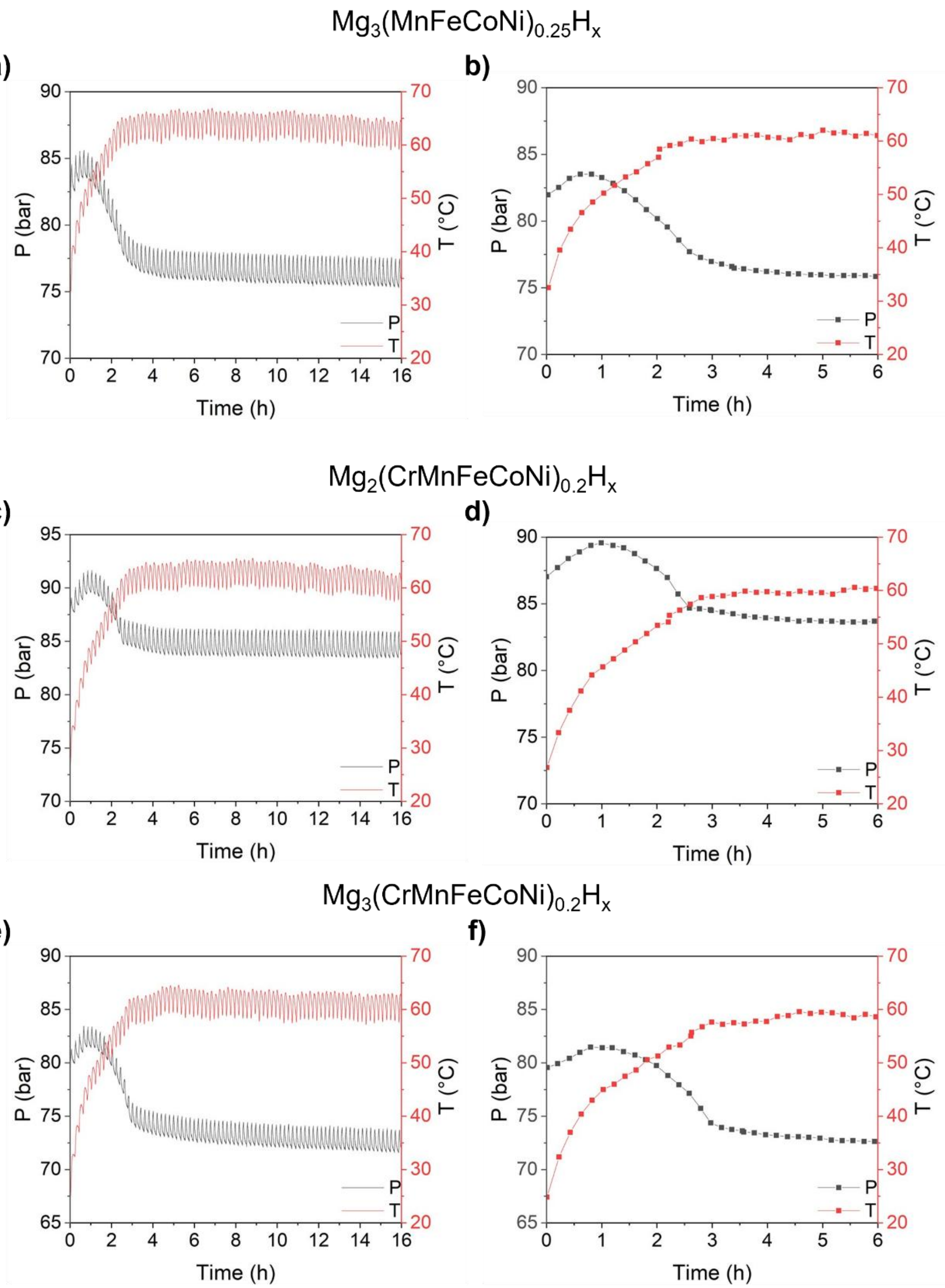

Figure S10: Pressure and temperature time-profile of the RBM synthesis process. Raw data of the full process (left) and p-T points collected at the end of each three-minutes cycle for $Mg_3(MnFeCoNi)_{0.25}H_x$ (**a,b**), $Mg_2(CrMnFeCoNi)_{0.2}H_x$ (**c,d**), $Mg_3(CrMnFeCoNi)_{0.2}H_x$ (**e,f**) .
